# Impact of screen exposure on pediatric vernal Keratoconjunctivitis: a survey during the COVID-19 pandemic in Italy

**DOI:** 10.1186/s13052-022-01253-2

**Published:** 2022-05-14

**Authors:** Marzio Masini, Giulia Brindisi, Mattia Giovannini, Elia Pignataro, Laura Di Grande, Cinzia De Libero, Marcella Nebbioso, Francesca Mori, Roberto Caputo, Anna Maria Zicari

**Affiliations:** 1 Department of Maternal Infantile and Urological Sciences, Division of Pediatric Allergology and Immunology, Sapienza University of Rome, 00185 Rome, Italy; 2 Pediatric Allergology Unit, Meyer Children’s University Hospital, Viale Pieraccini 24, 50139 Florence, Italy; 3 Clinical Ophthalmology Unit, Meyer Children’s University Hospital, Viale Pieraccini 24, 50139 Florence, Italy; 4 Department of Sense Organs, Sapienza University of Rome, 00185 Rome, Italy

**Keywords:** Vernal keratoconjunctivitis (VKC), Children, SARS-CoV-2, COVID-19, Lockdown, Survey, Screens

## Abstract

**Background:**

The SARS-CoV-2 outbreak pushed the Italian government to start a strict lockdown, replacing school attendance with long-distance learning. This caused reduced exposure to sunlight but increased exposure to screens. Vernal keratoconjunctivitis (VKC) is a chronic inflammatory ocular condition in which exposure to light plays a cardinal role. We conducted an online survey to evaluate the impact of screen exposure on children with VKC during the COVID-19 lockdown.

**Methods:**

We performed a survey-based observational study, asking patients followed at the Allergology clinics of Meyer Children’s University Hospital in Florence and of Policlinico Umberto I in Rome to provide grading on 6 subjective ocular clinical manifestations presented during the lockdown and to give an estimate of their hours/day of screen exposure.

**Results:**

Mean scores of signs and symptoms increased homogeneously when studying patients exposed to longer screen time. When comparing scores collected in 2019 to those in 2020, there was not a significant reduction in clinical manifestations, although the situation differed between the two centers due to geographical differences in sunlight exposure.

**Conclusion:**

During the lockdown, there was a reduction in sunlight exposure but conversely an increase in the time spent in front of screens that correlated with the worsening of VKC signs and symptoms in direct proportion to the hours/day of screen exposure. Our results also showed a statistically significant difference in the relative impact of long-distance learning on VKC clinical manifestations in the different Italian regions.

## Background

In March 2020, a disease caused by the novel coronavirus SARS-CoV-2 was declared a pandemic by the World Health Organization with a rapid spread from China all over the world [1]. Italy is one of the hardest-hit countries with a total of 5,172,185 confirmed cases and 133,967 reported deaths, at the time of writing [2].

SARS-CoV2 causes a broad spectrum of clinical manifestations leading, in critical cases, to severe acute respiratory syndrome with intensive care hospitalization [3, 4]. However, COVID-19 in children often presents mild symptoms not requiring hospitalization [5]. As a consequence of the rapid viral spread, governments in Italy and other countries announced strict measures to flatten the contagion curve. In Italy, this period lasted from the 11th of March 2020 to the 3rd of May 2020 with the obligation to stay at home, except for urgent medical reasons; free access to outdoor recreational activities was only granted from June 15th. This had severe consequences on the entire population’s well-being as far as mental health [6–10], nutrition [11, 12] and sleep quality [11, 13, 14] were concerned. Patients affected by chronic diseases, like VKC, were followed up via phone calls or emails [15].

VKC is a rare [16] chronic conjunctivitis that affects children and usually resolves in puberty. Typical signs and symptoms are shared with allergic conjunctivitis (AC), and include photophobia, redness, itching and tearing; the common drugs used in AC, such as antihistamines, are usually ineffective against these symptoms in cases of VKC.

Many hypotheses have been made concerning VKC aetiology, but the actual mechanism supporting the chronic inflammation in ocular conjunctiva still remains debated. Besides the autoimmune and allergic aetiology, a recent and intriguing hypothesis has considered the role of oxidative stress in keeping chronic conjunctival inflammation active [17]. It is also worth remembering that VKC is a disease in which sun exposure, humidity/temperature, and weather conditions seem to play a role, especially in regards to its prevalence and, to a lesser extent, the severity of symptoms. In fact, although the scarcity of epidemiological data makes the claim difficult [18, 19], it cannot be denied that in daily clinical practice there is usually a slightly increasing prevalence gradient from northern to southern regions.

In the management of VKC, it is essential to start early treatment with immunosuppressant agents. Nevertheless, topical corticosteroids are frequently associated with the appearance of cataracts and glaucoma. Therefore, although there is no uniformity in the definition of disease severity, patients benefit from the use of other drugs, such as cyclosporine A or tacrolimus eye drops, in order to reduce ocular inflammation and prevent long-term ocular damage [20–28].

In Italy, during the lockdown, the widespread closure of schools marked the beginning of long-distance learning. Children and teenagers experienced for the first time remote teaching at home, with the use of smartphones, computers and tablets for many hours/day, resulting in several physical and psychological consequences [29]. Light and especially sunlight play the primary role in worsening VKC clinical manifestations. We would have expected that the reduced sunlight exposure due to lockdown would have greatly benefitted our patients. On the contrary, in our daily clinical practice, we noticed that our patients had similar or worse signs and symptoms than the previous years.

To the best of our knowledge, at the time of writing, no studies reported data on clinical manifestations in children affected by VKC during the COVID-19 lockdown. Moreover, data on the effects of using digital screens on VKC signs and symptoms are scarce and usually hard to quantify. Long-distance learning on a national scale represented a unique opportunity to explore the effects of regular and well-quantifiable exposure to screens on several pediatric patients affected by VKC. Thus, we conducted an online survey to evaluate the impact of long-distance learning in children with VKC during the COVID-19 lockdown and the period of strict government-issued outdoor activity regulations.

## Materials and methods

We performed a retrospective survey-based observational study enrolling children with VKC, aged 5 to 17 years, from the Allergy Unit of Meyer Children’s University Hospital in Florence and from the Pediatric Allergology Clinic of the Policlinico Umberto I in Rome. Inclusion criteria were the following: diagnosis of VKC made at least 1 year before enrollment based on clinical history and ocular examination with signs and symptoms of VKC carried out by an expert physician. Exclusion criteria were the presence of other chronic ocular or systemic diseases.

Regarding clinical grading/history, each patient’s parent was asked to complete an online questionnaire focused on ocular signs and symptoms (itching, photophobia, tearing, foreign body sensation and burning sensation), which were scored as previously reported [21, 22], referring to the period of March – June 2020 (strict lockdown ended on May 4th 2020, but long-distance learning continued until the end of the school year, which in Italy was between June 6th and 16th depending on the region; important limitations were still in place in June 2020). Each variable was graded: 0 = no symptoms; 1 = mild symptoms; 2 = moderate symptoms; 3 = severe symptoms. Each patient’s parent was also asked to give an estimate of the amount of time spent in front of a screen (e.g., tablet, computer) due to long-distance learning, and patients were divided into 4 groups: less than 2 h/day, 2–4 h/day, 4–6 h/day, and more than 6 h/day of screen exposure. As accessory data, we asked the patient’s parent to grade the same clinical manifestations for the same period (March – June) of the previous year (2019) to get an idea of the change in the symptoms between 2019 and 2020. Maintenance therapy was also registered, as well as demographic characteristics and the presence of atopic and non-atopic comorbidities.

All data were analyzed with the use of IBM SPSS version 26 statistical software (IBM, Armonk, New York, United States of America). The Student t-test for paired samples was used for the comparison of mean scores between different times of exposure. *P*-values less than 0.05 were considered statistically significative.

## Results

Questionnaires were sent to a total of 248 families of VKC patients followed by the two centers. Specifically, 102 questionnaires were sent to patients from the Roman center and 146 were sent to patients from the Florence center. A total of 161 VKC patients responded and were recruited into the study: 98 patients (60.87%) from the allergy unit of the Meyer Children’s University Hospital (Florence) and 63 patients (39.13%) from the Pediatric Allergology Clinic of Policlinico Umberto I (Rome) with a diagnosis of VKC. The mean age was 10.9 ± standard deviation (SD) 2.97 years (range 5–17 years, M:F = 3:1). Age and sex did not differ statistically between the two centers (age t test *p*-value: 0.877; sex chi-squared test p-value: 0.892). In detail, the mean age at onset of VKC was 7.05 ± SD 2.73 years. Of these patients, 47.2% reported 1 or more allergic comorbidities, 35 (22%) reported rhinitis, 24 (14.9%) reported asthma, 38 (23.6%) reported atopic dermatitis, 8 (4.96%) reported food allergy. As previously mentioned, we divided our patients by the average number of hours of exposure to screens due to long-distance learning, forming 4 “Exposure groups”: less than 2 h/day (50 patients, or 31% of total), 2–4 h/day (63 patients, 39% of total), 4–6 h/day (28 patients, 17.39% of total) and more than 6 h/day (12 patients, 7.5% of total). The “Less than 2 hours/day” exposure group was not included in this part of the study since that kind of exposure to long-distance learning, the focus of our study, was not deemed significant. Screen exposure not related to long-distance learning was considered difficult to quantify and was not the focus of our study.

Data results are summed up in Tables 1 and 2.

**Table 1:** Characteristics of the study population

Characteristics (***n*** = 161)	
Age (years)	10.9 ± 2.97
Females	41 (25.46%)
Age of onset (years)	7.05 ± 2.73
Patients with atopic comorbidities - total	76 (47.2%)
Rhinitis	35 (22%)
Asthma	24 (14.9%)
Atopic dermatitis	38 (23.6%)
Food Allergy	8 (4.96%)
Exposure groups	
0–2 h/day	50 (31%)
2–4 h/day	63 (39%)
4–6 h/day	28 (17.39%)
More than 6 h/day	12 (7.5%)

Data reported as N (%) or mean ± standard deviation

**Table 2:** Symptom scores by exposure group

		Itching	Photophobia	Tearing	Foreign Body Sensation	Burning
March–June 2020: **2-4 h/day** (*n* = 63)	Mean	1.1111	1.1269	0.9206	0.9047	1.1269
	SD	0.9000	0.9586	0.9384	0.9624	1.0394
	Total	5.1904				
March–June 2020: **4-6 h/day** (*n* = 28)	Mean	1.0714	1.3571	1.2143	0.9643	1.1785
	SD	0.8997	1.0959	0.7868	0.9222	0.9833
	Total	5.7857				
March–June 2020: **> 6 h/day** (*n* = 12)	Mean	1.4166	1.3333	1.4166	1.4166	1.5833
	SD	0.7929	1.1547	0.9003	0.9962	0.5149
	Total	7.1666				
**Pearson correlation coefficient** (screen exposure vs symptoms)		0.8087	0.8151	0.9944	0.9142	0.9130

Mean symptom scores for children exposed to long-distance learning, divided into exposure groups; Pearson correlation coefficients between symptom scores and screen exposure

*SD* Standard Deviation

**Fig. 1:**
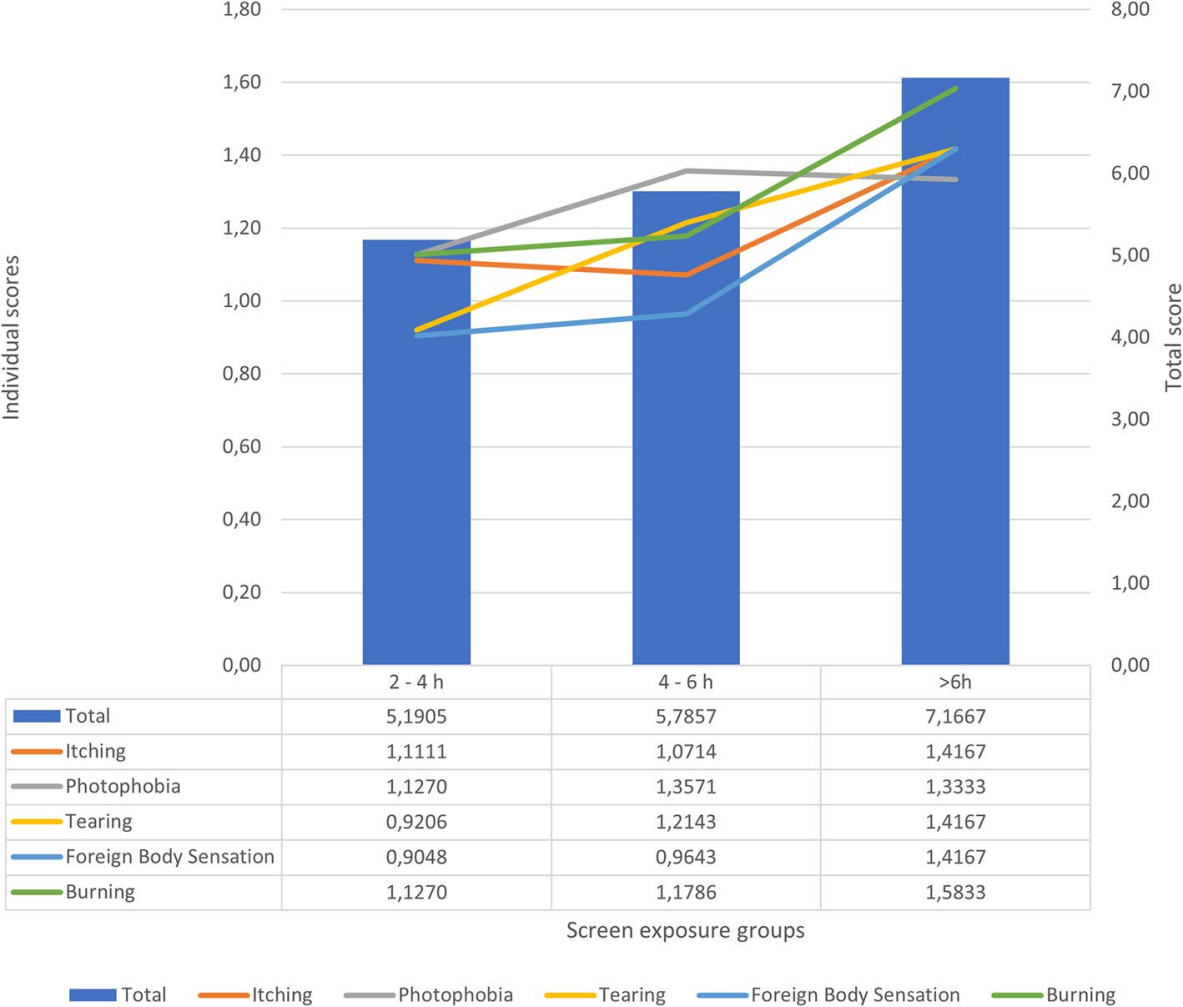
Symptoms scores. Bar graph and table comparing individual and total symptom scores across screen exposure groups

A graph showing trends in clinical manifestation scores can be seen below. (Fig. 1):

We found mean scores of signs and symptoms to homogeneously increase when studying patients exposed to progressively longer screen times. This was particularly true when comparing burning, foreign body sensation and tearing, which saw steady increases from the 2–4 h/day group to the more than 6 h/day group.

Itching was found to be stable between the 2–4 and 4-6 h/day groups, and photophobia seemed to be slightly less pronounced in the “more than 6 hours/day” group when compared to the “4-6 h/day” group. However, the t-test between those two groups was not significant (*p* = 0.423), so we can interpret this finding as photophobia not being statistically different between the 4-6 h/day and the more than 6 h of screen time/day populations.

Nonetheless, while evaluating each clinical manifestation, we saw an increase in severity when comparing the group with the least exposure to the group with the most exposure. However, probably due to the low number of patients in the “More than 6 hours/day” group, the differences were not statistically significant for itching and photophobia scores, which showed, respectively, T-test *p* values of 0.1240 and 0.2848. All the other symptoms had significantly higher mean scores in the “More than 6 h/day” group versus the “2–4 h/day” group, with p values of 0.050 for “Tearing”, 0.0453 for “Foreign body sensation” and 0.0140 for “Burning”.

Moreover, we asked parents to estimate their children’s eye signs and symptoms looking back at the same period we explored in the previous paragraphs (March–June) of the previous year (2019), so as to get an approximate idea of how the lockdown impacted VKC clinical manifestations, with a positive influence (less sunlight exposure) or negative influence (more digital screens exposure) (Table 3).

**Table 3:** Mean score comparison (2019 vs 2020) - pooled results (Florence + Rome)

	Itching	Photophobia	Tearing	Foreign Body Sensation	Burning
Mean 2019 (SD)	1.3478 (±0.9438)	1.4968 (±0.9754)	1.2298 (±0.9370)	1.2670 (±0.9985)	1.3478 (±0.9569)
Mean 2020 (SD)	1.2360 (±0.9186)	1.3229 (±1.0037)	1.1428 (±0.9209)	1.0683 (±1.0007)	1.2484 (±0.9939)
T Test *p*-values	0.0947	0.0067	0.1449	0.0040	0.1446

*SD* Standard deviation, *T Test* Student T test

Photophobia and foreign body sensation showed a decrease between the same periods of 2019 and 2020, with a statistically significant difference (T-test *p*-values respectively 0.006 and 0.004); all the other signs and symptoms were not reported as significantly different. When separately evaluating the scores reported by the two centers, different data were demonstrated (Table 4).

**Table 4:** Mean scores comparison (2019 vs 2020) – individual results

		Itching	Photophobia	Tearing	Foreign Body Sensation	Burning
**Florence**	**Mean 2019 **(SD)	1.2449 (±0.897)	1.3673 (±0.901)	1.2143 (±0.864)	1.2041 (±0.984)	1.2347 (±0.928)
	**Mean 2020** (SD)	1.2551 (± 0.945)	1.3775 (± 0.969)	1.2551 (± 0.889)	1.1734 (± 1.016)	1.3367 (± 0.963)
	**T Test** ***p*** **-values**	0.1973	0.2944	0.6942	0.2507	0.00003
**Rome**	**Mean 2019** (SD)	1.5079 (± 0.998)	1.6984 (± 1.057)	1.2539 (± 1.047)	1.3650 (± 1.02)	1.5238 (± 0.981)
	**Mean 2020** (SD)	1.2063 (± 0.883)	1.2381 (± 1.058)	0.9682 (± 0.95)	0.9047 (± 0.962)	1.1111 (± 1.033)
	**T Test** ***p*** **-values**	0.0190	3.28E-06	0.0014	7.12E-05	0.0003

*SD* Standard deviation, *T Test* Student T test

Children referring to Meyer Children’s University Hospital reported a slight worsening of their signs/ symptoms during the lockdown, despite being exposed to long-distance learning with a similar distribution to their peers of Policlinico Umberto I (mean screen exposure – Policlinico Umberto I: 3.06 h/day; Meyer: 3 h/day, T-test *p*-value: 1). However, the differences were not statistically significant except when considering burning sensation scores.

Instead, children from the latter center reported a statistically significant reduction in the severity of signs and symptoms during the lockdown period compared with the same period of the previous year.

It is important to acknowledge that the two Centers follow patients from several different regions of Italy, with Meyer’s Children’s Hospital gathering patients mostly from central and northern regions. The average latitude of places of residence of patients from the Tuscan center was 43.712° (SD ±2.06°), while patients from the Roman center were from cities, on average, from southern regions, averaging a latitude of 41.910° (SD ±0.211°). The difference was statistically significant (T test *p* value 3.597 × 10^− 7^).

## Discussion

To the best of our knowledge, this is the first survey that evaluated the impact of exposure to screens during the COVID-19 lockdown on VKC clinical manifestations in a pediatric population.

The management of several diseases during home quarantine has changed and adapted to this critical period where the priority of the hospitals has been the management of emergencies. For this reason, the use of surveys has been useful to understand the impacts and consequences of home quarantine on signs and symptoms in several pathologies, as well as VKC.

VKC is not, like allergies, a risk factor for the susceptibility to SARS CoV- 2 infections, and neither is their treatment; for this reason, the standard treatment should be continued in VKC as well as in allergic pathologies [30–32]. VKC is a chronic inflammatory ocular pathology in which photophobia is one of the most troublesome clinical manifestations, together with foreign body sensation, itching and tearing that are also present in AC [17].

During the lockdown, there was a reduction in exposure to sunlight, but conversely, the time spent in front of PCs, TVs and video games increased [33]. During the lockdown, long-distance learning has been instituted for children over the age of 5, using video-conference platforms. This had the drawback of exposing children and adolescents to a critical load of screen time. Indeed, some of our patients had to regularly sit in front of screens for 6 or more hours per day.

Establishing controlled exposure to screens allowed us to evaluate more accurately than before the effect of different loads of light exposure from screens on a subset of children, as VKC patients, for whom light is a trigger of clinical manifestations.

Consistent with this situation, results of our study have shown a general trend of worsening of VKC clinical manifestations, such as tearing, foreign body sensation and burning, when comparing the “2–4 h/day” group to the “more than 6 hours/day” group. The T-test analysis showed a statistically significant difference between the group with the minimum exposure compared to the group with the most exposure (“more than 6 hours of screen time/day”) to screens.

Instead, the symptom of itching was found to be stable between the 2–4 and 4-6 h/day groups and the symptom photophobia was apparently less severe in the “more than 6 hours/day” group when compared to the “4-6 h/day” group, but the statistical analysis found no statistical significance.

A strong Pearson correlation coefficient has shown a high correlation between screen exposure and all VKC symptoms, such as itching, photophobia, tearing, foreign body sensation, and burning.

Moreover, considering the comparison between 2020 and 2019 in the same period (March–June), only photophobia and foreign body sensation showed a decrease in 2020 in comparison with the previous year, with a statistically significant difference (T-test *p*-values ​​respectively 0.006 and 0.004). In contrast, all other signs and symptoms were not found as significantly different.

When considering Rome and Florence separately, the statistical analysis revealed further information. Referring to Policlinico Umberto I Hospital, we found a statistically significant reduction in the severity of symptoms during home quarantine in 2020 compared to the same period of the previous year. Instead we have seen a mild worsening of VKC symptoms among patients from Meyer Children’s University Hospital during the lockdown period in 2020 compared to the same period of 2019, but with poor statistical significance: only for the clinical manifestation of burning the difference between 2020 versus 2019 reached statistical significance.

These results could be explained by the different weather and latitudes of the two centers. Sunlight exposure is more abundant in southern regions, so patients referring to Policlinico Umberto I probably experienced a relative reduction of light exposure during the lockdown compared to the previous year, even considering the “Mandatory” screen light exposure required by long-distance learning. This might have played a role in the reported improvement in signs and symptoms by children referring to this center. In northern regions, sunlight is less abundant, so the reduction in sunlight exposure is relatively less significant when compared to the increase in screen exposure, resulting in a slight worsening of VKC signs and symptoms. To the best of our knowledge, no studies regarding the specific influence of latitude on VKC symptoms have been carried out yet.

In literature, several studies have been conducted considering allergic pathologies and in particular allergic conjunctivitis during lockdown [1, 31, 34], but to the best of our knowledge, no studies are reporting VKC symptoms during it.

The study conducted by Walaa Al-Dairi et al. analyzed the impact of lockdown on patients’ quality of life with allergic conjunctivitis, and it was reported, unlike our results, that there was no important effect on the quality of life of allergic conjunctivitis during the COVID-19 lockdown [35].

Leonardi et al., in accordance with the EAACI guidelines [36], provided indications in the management of seasonal (SAC) and perennial (PAC) allergic conjunctivitis, VKC and atopic keratoconjunctivitis (AKC), highlighting that the treatment options for SAC/PAC, VKC and AKC were still valid also during COVID-19 outbreak [30].

### Limitations

This study presents some limitations; first of all, it was based on online surveys, that were retrospectively collected in December 2020. Parents’ recollections of previous clinical signs and symptoms could have been affected, and we couldn’t use medical records referring to 2019 since they focused more on clinical ophthalmologic signs rather than parents’ perceptions of symptoms, which was the focus of our inquiry. Another limitation was that we used a non-standardized questionnaire (however, to the best of our knowledge, standardized questionnaires on this topic are not available). Selection bias also should be considered since people more interested in the SARS-CoV-2 outbreak may have been more likely to answer the survey. Moreover, our study focused exclusively on the better quantifiable screen exposure represented by long-distance learning, and it didn’t focus on less quantifiable exposure, like videogames or smartphone use, or natural light exposure from sources like balconies and private gardens. Lastly, the sample size is relatively small. Thus, further studies with a larger sample size should be desirable in the future to shed light on this specific topic.

## Conclusions

Screen exposure to long-distance learning correlates with the worsening of VKC clinical manifestations in proportion to the number of hours/day of exposure. The establishment of long-distance learning and the regular screen exposure prescribed by schools made it possible to carry out a dedicated investigation and ascertain this correlation. In Italy, during the COVID-19 lockdown, there was a reduction of the exposure to sunlight but conversely an increase in the time spent in front of screens. This could explain the general trend of increasing VKC signs and symptoms in our pediatric population. Therapy should be continued as well as the close monitoring of clinical manifestation in children with VKC participating in long-distance learning.

## Data Availability

The data that support the findings of this study are available on request from the corresponding author. The data are not publicly available due to privacy or ethical restrictions.

## Abbreviations

VKC: Vernal keratoconjunctivitis
AC: Allergic conjunctivitis
SAC: Seasonal allergic conjunctivitis
PAC: Perennial allergic conjunctivitis
AKC: Atopic keratoconjunctivitis
SD: Standard deviation
